# When Real-World Outcomes Do Not Meet the Results of Clinical Trials: Transfemoral Transcatheter vs. Surgical Aortic Valve Replacement in an Intermediate-Age Population (The Outstanding Italy Study)

**DOI:** 10.3390/jcm14103471

**Published:** 2025-05-15

**Authors:** Marco Ranucci, Lidia Staszewsky, Massimo Cartabia, Mauro Tettamanti, Vito Lepore, Fabio Robusto, Antonio Clavenna, Antonio D’Ettorre, Eloisa Arbustini, Damiano Baldassarre, Maria Teresa La Rovere, Matteo Montorfano, Gianfranco Parati, Roberto F. E. Pedretti, Giuseppe Maria Raffa, Francesco Santini, Giulio Stefanini, Maurizio Volterrani, Ida Fortino, Lucia Bisceglia, Lorenzo Menicanti, Roberto Latini

**Affiliations:** 1 Department of Cardiovascular Anesthesia and Intensive Care, IRCCS Policlinico San Donato, 20097 Milan, Italy; 2 Laboratory Clinical Research in Brain and Cardiovascular Injury, Department of Acute Brain and Cardiovascular Injury, Istituto di Ricerche Farmacologiche Mario Negri IRCCS, 20156 Milan, Italyroberto.latini@marionegri.it (R.L.); 3 Laboratory of Geriatric Neuropsychiatry, Department of Health Policy, Istituto di Ricerche Farmacologiche Mario Negri IRCCS, 20156 Milan, Italymauro.tettamanti@marionegri.it (M.T.); 4 Laboratory of Medical Research and Consumer Involvement, Department of Public Health, Istituto di Ricerche Farmacologiche Mario Negri IRCCS, 20156 Milan, Italy; vito.lepore@marionegri.it; 5 Puglia Health System, Medonline-Statte, ASL-TA, 74010 Taranto, Italy; fabiorobusto@libero.it (F.R.);; 6 Scientific Department, Centre for Inherited Cardiovascular Diseases, Fondazione IRCCS Policlinico San Matteo, 27100 Pavia, Italy; antonio.clavenna@marionegri.it (A.C.); e.arbustini@smatteo.pv.it (E.A.); 7 Centro Cardiologico Monzino, IRCCS, 20138 Milan, Italy; damiano.baldassarre@cardiologicomonzino.it; 8 Department of Medical Biotechnology and Translational Medicine, Università degli Studi di Milano, 20129 Milan, Italy; 9 Department of Cardiology, Istituti Clinici Scientifici Maugeri, Montescano Institute-IRCCS, 27040 Montescano, Italy; mariateresa.larovere@icsmaugeri.it; 10 School of Medicine, Vita-Salute San Raffaele University, 20132 Milan, Italy; montorfano.matteo@hrs.it; 11 Interventional Cardiology Unit, IRCCS San Raffaele Scientific Institute, 20132 Milan, Italy; 12 Istituto Auxologico Italiano, S. Luca Hospital, University of Milan-Bicocca, 20126 Milan, Italy; gianfranco.parati@unimib.it; 13 Department of Medicine and Surgery, University of Milano-Bicocca, Piazza dell’Ateneo Nuovo, 20126 Milan, Italy; 14 School of Medicine and Surgery, University of Milano Bicocca, 20126 Milano, Italy; robertofrancoenrico.pedretti@multimedica.it; 15 Cardiovascular Department, IRCCS MultiMedica, 20099 Sesto San Giovanni, Italy; 16 Department for the Treatment and Study of Cardiothoracic Diseases and Cardiothoracic Transplantation, IRCCS-ISMETT, 90127 Palermo, Italy; graffa@ismett.edu; 17 Cardiac Surgery Unit, Department of Precision Medicine in Medical Surgical and Critical Area (Me.Pre.C.C.), University of Palermo, 90133 Palermo, Italy; 18 Cardiac Surgery, Ospedale Policlinico San Martino, University of Genoa, 16126 Genoa, Italy; francesco.santini@unige.it; 19 IRCCS Istituto Clinico Humanitas, 20089 Rozzano, Italy; giulio.stefanini@hunimed.eu; 20 Cardiopulmonary Department, IRCCS San Raffaele Roma, 00166 Rome, Italy; maurizio.volterrani@sanraffaele.it; 21 Exercise Medicine and Science, San Raffaele Open University, 00166 Rome, Italy; 22 Puglia Health System, Agenzia Regionale Strategica per la Salute ed il Sociale, AReSS Puglia, Area Epidemiologia e Care Intelligence, 70121 Bari, Italy; ida_fortino@regione.lombardia.it; 23 Laboratory for Pharmacoepidemiology, Department of Public Health, Istituto di Ricerche Farmacologiche Mario Negri IRCCS, 20156 Milan, Italy; l.bisceglia@aress.regione.puglia.it; 24 Department of Cardiac Surgery, IRCCS Policlinico San Donato, 20097 Milan, Italy; lorenzo.menicanti@grupposandonato.it

**Keywords:** aortic valve stenosis, transcatheter aortic valve implantation, surgical aortic valve replacement, outcome

## Abstract

**Objective**: Aortic valve stenosis is the most common valvular heart disease in the elderly, and its treatment may be either surgical (SAVR) or transcatheter (TAVI). Although age is one of the main determinants of the therapeutic choice, current guidelines leave a “discrepancy area” between 65 and 75 years, with the American guidelines allowing TAVI for patients older than 65 years, while the European guidelines consider TAVI for patients older than 75 years. The present study addresses the outcomes of SAVR vs. TAVI in a real-world population aged 65 to 80 years, that is, one largely inclusive of the discrepancy area. **Methods**: This is a retrospective registry study based on data retrieved from administrative health databases of two large Italian regions (Lombardy and Puglia). Patients aged 65 to 80 years receiving either SAVR or a TAVI between 2018 and 2021 were selected. SAVR and TAVI outcomes (death, cardiac and non-cardiac events) were compared using a propensity-matching analysis, with a follow-up of 2 to 5 years and mortality as the primary outcome. **Results**: After propensity matching, two groups of 786 patients were compared in Lombardy and two groups of 321 patients were compared in Puglia. In both regions, at the end of follow-up, mortality was significantly (*p* < 0.001) lower in SAVR vs. TAVI (24.6% vs. 47.2% in Lombardy and 18.1% vs. 44.1% in Puglia). **Conclusions**: Our results are in contrast with the randomized controlled trials showing equivalence or even the superiority of TAVI vs. SAVR, but in agreement with other registry studies based on real-world data. With respect to the randomized controlled trials, the main difference is a better outcome in SAVR. Caution should be applied in addressing patients < 80 years with TAVI unless SAVR is contraindicated.

## 1. Introduction

With aging populations, the prevalence of aortic valve stenosis (AS) is continuously increasing [1]. Aortic valve replacement (AVR) can be performed with open heart surgery (SAVR) or transcatheter aortic valve implantation (TAVI), with the second being the procedure of choice in patients at high surgical risk [2].

However, in selecting SAVR or TAVI, there is still a “gray area” represented by patients at an intermediate age (between 65 and 80 years), and, in particular, there are discrepancies between the European [2] and American [3] guidelines, recently analyzed in various studies [4,5,6]. In the absence of specific contraindications, the European guidelines’ age limit for TAVI is <75 years, and in the US guidelines it is <65 years. Previous studies comparing the two procedures in this segment of patients did not report conclusive results [7,8,9,10]. Data from registries from ≤80 years old patients showed that long-term mortality was higher after TAVI than after SAVR [11,12].

The OUTcomes evaluation of current therapeutic STrategies for severe Aortic valve steNosis anD the agING population in ITALY (OUTSTANDING ITALY) registry was established with the aim of deepening research on outcomes after the two different procedures of AVR, comparing the incidence of fatal and non-fatal events in patients aged 65 to 80 years old undergoing SAVR or TAVI for severe aortic stenosis in a large real-world population. The patient population considered is representative of a possible area of uncertainty in the decision to refer a patient for SAVR or TAVI. Whereas at <65 years it is quite rare to choose TAVI, at >80 years this procedure is very often considered; additionally, the population includes the 65–75 years range, where there is a discrepancy in the indications reported by the existing guidelines [2,3].

At the time when the study was planned, there were only randomized controlled trials (RCTs) comparing the outcome of SAVR vs. TAVI, so the novelty of the study is in its addressing of a real-world scenario based on administrative data and, hence, being reliable for its primary outcome (mortality). There are presently other registry studies that will be discussed. However, our study is the only one selecting 65–80 year old patients, who represent a possible gray area inclusive of the guidelines-based discrepancy area.

## 2. Materials and Methods

The OUTSTANDING ITALY registry was established in 2018 and started enrolling patients at the end of 2018. 

The registry was established within the Cardiac Network of the Italian Clinical Research Hospitals (IRCCSs), funded by the Italian Ministry of Health. There are different branches within the OUTSTANDING registry. The main data collection is prospective and includes an assessment of quality of life before and after a procedure, with a 48-month follow-up; another branch explores neurocognitive function before and soon (3 months) after the procedure. Finally, retrospective data collection was subsequently admitted and is the basis of the present study. The study was approved by the local ethics committee of San Raffaele Hospital (OSR 14/12/2017, protocol number 298/2017) and was registered at clinicaltrials.gov (NCT 05778773). The patients in the retrospective cohort gave general consent for the use of their data in an anonymous form and for scientific purposes, and the retrospective data do not overlap with the prospectively collected data, which will be analyzed separately in a different study. The present study was based on data made available upon formal agreements between the participating IRCCSs (Istituto di Ricerche Farmacologiche Mario Negri) and the Health Systems of two large Italian regions.

The data included in the analysis to characterize the patients were checked for completeness; no missing imputation was therefore needed. The completeness of the registration of the patients was checked by accessing the registry of the Coordinating Center, Policlinico San Donato; more than 95% of the patients were retrieved from the Administrative Regional Database. This finding reassured us against the under-recording of cases. The severity of the reported outcomes makes under-reporting unlikely. The study outcomes were major events that required hospitalization with a subsequent ICD-9 codification. Moreover, since event codification is associated with a hospital remuneration system for care activities by the National Health Service, it may be subject to possible centralized controls, so both under-reporting and mis-reporting are unlikely.

### 2.1. Data Source

This study used the linkable administrative health databases of two Italian regions, Lombardy and Puglia. Data were available from 2018 to 2022 for about 10 million inhabitants of Lombardy and 4 million of Puglia. In particular, data were collected in the following databases and acquired through anonymized record-linkage procedures: the Regional Assistance Registry, Pharmaceutical Prescriptions, Hospital Discharge Forms, Passive mobility and Exemptions for pathology.

### 2.2. Study Population

We included in the present analysis patients hospitalized between 1 January 2018 and 31 December 2021 aged 65 to 80. Comorbidities for the 5 years before the index intervention were collected using hospital records. 

The criteria used to define comorbidities prior to the index event are reported Appendix A.

All subjects were followed-up with for a maximum of 5 years after the date of the aortic valve replacement procedure until 31 December 2022.

### 2.3. Study Outcomes

The main objective of the study was to compare the short- and long-term risks of all-cause mortality in the TAVI and SAVR cohorts of patients with AS aged 65 to 80 years.

The secondary endpoints were the short- and long-term incidence of cardiovascular and non-cardiovascular events. Short- and long-term complications, cardiovascular or non-cardiovascular, are listed in Appendix B.

All events were considered as the primary diagnosis performed during emergency room or hospital admissions.

### 2.4. Propensity Score Matching

In order to make the characteristics of the two populations comparable, a match-based analysis was carried out using one-to-one propensity score matching with the nearest-neighbor method. 

The adequacy of the propensity score matching was assessed by the standardized mean differences in the following patient characteristics post-matching: age, sex, year of AVR (two categories: 2018–2019, 2020–2021), the region of each subject where the AVR was performed and comorbidities diagnosed in the 5 years preceding the date of the index surgery, see (ANNEX 1). Good balance is conventionally set at a standardized mean difference of −0.1 to 0.1.

### 2.5. Statistical Analysis

Data are expressed as mean (standard deviation) or median (quartiles) for continuous variables and number (percentage) for categorical variables. 

The outcomes of interest were analyzed in the period 0–30 days post-AVR with a logistic regression model in order to estimate the odds ratios (ORs), while in the period beyond 30 days, Kaplan–Meier survival curves and the Cox model were used to estimate hazard ratios (HRs) and their respective 95% confidence intervals (CIs). This choice was made because when using the Cox model for the entire observation period, from 0 to 1825 days, the assumption of the proportionality of the HRs, required by the model, was not fulfilled. Consequently, the observation period was divided into two time sections: (a) 0–30 days; (b) 31–1825 days.

The multivariable analyses were adjusted for the following variables: sex, age, year of surgery (two categories) and all study comorbidities (ANNEX 1).

For non-fatal events after 30 days, to confirm the results from the multivariable Cox model, an adjustment for competing risk of death was performed using the Fine–Gray univariate and multivariate model including the same covariates as in the Cox model. In addition, the subjects were further characterized by the Drug Derived Complexity Index (DDCI), a validated index of illness severity derived from drug prescriptions and able to stratify the general population according to their risk of death, unplanned hospital admission and readmission [13], which was included as a covariate in the Cox multivariable model as a sensitivity analysis.

The rate of hospitalization or emergency department visits for COVID-19 during follow-up was assessed; the risk of all-cause mortality was also analyzed in subjects with and without COVID-19. The 3 sensitivity analyses described above were conducted only on data from the Lombardy region.

All the *p* values were two-tailed and considered statistically significant when <0.05. SAS statistical software version 9.04 (SAS Institute Inc., Cary, NC, USA) was used for all the analyses.

## 3. Results

**Figure 1:**
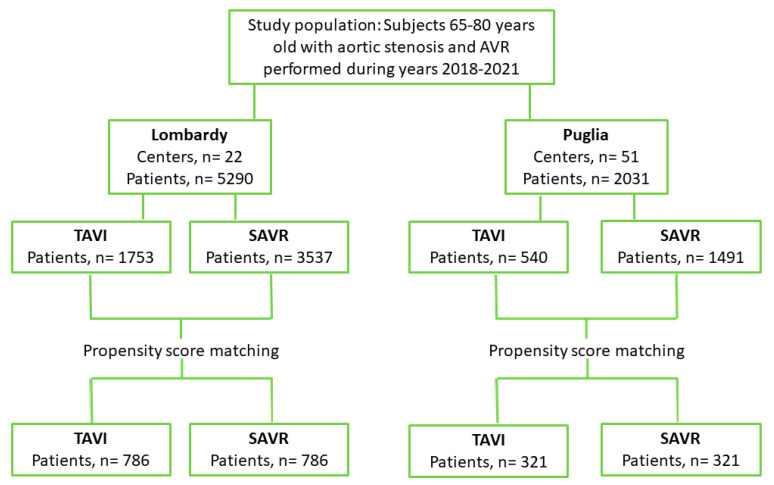
Study flow-chart. SAVR: surgical aortic valve replacement; TAVI: transcatheter aortic valve implantation.

Between 1 January 2018 and 31 December 2021, 5290 subjects with AS and valve replacement were identified in Lombardy, and 2031 were identified in Puglia (Figure 1). Of these, 1753 (33%) in Lombardy and 540 (27%) in Puglia underwent TAVI, while 3537 (67%) and 1491 (73%) underwent SAVR. The main characteristics of the population are shown in Table 1.

The mean age of patients undergoing TAVI was 76.3 ± 3.5 and 76.5 ± 3.6 years in Lombardy and Puglia, respectively, while those undergoing SAVR were significantly younger, 73.4 ± 4.3 vs. 73.6 ± 4.2 years, respectively (*p* < 0.0001 for both regions, Table 1). The frequency of comorbidities in both studied regions was significantly higher in the group of subjects undergoing TAVI than SAVR (*p* < 0.0001 for all factors, Table 1).

Compared to the two-year period of 2018–2019, in the years 2020–2021, the incidence of TAVI interventions increased by 19% in Lombardy and 25% in the Puglia region, while the incidences of SAVR decreased, respectively, by 30% and 31%.

The median [Q1–Q3] observation time was 787 [513–1213] days in the TAVI group and 1128 [648–1468] days in the SAVR group (*p* < 0.0001) in Lombardy and 785 [539–1124] days in the TAVI group and 1079 [495–1426] days in the SAVR group (*p* < 0.0001) in Puglia.

### 3.1. Propensity-Score-Matched Cohorts

After propensity score matching, 1572 patients were identified in Lombardy and 642 in Puglia (Table 2).

The baseline characteristics results aligned between the TAVI and SAVR cohorts from both regions (Supplementary Figure S1), with the exception of pulmonary embolism (only one subject in all the matched subpopulations) and oncological disease.

The median [Q1–Q3] observation time was 858 [549–1304] days in the TAVI group and 969 [592–1416] days in the SAVR group (*p* < 0.0001) in Lombardy and 809 [555–1176] days in the TAVI group and 809 [555–1146] days in the SAVR group (*p* < 0.0001) in Puglia.

### 3.2. Primary Outcome in Propensity-Score-Matched Cohorts

At the end of follow-up, the mortality rates in the propensity-score-matched cohorts in Lombardy were 47.2% in the TAVI cohort and 24.6% in SAVR, and in Puglia, 45.3% and 20.7%, respectively. The Kaplan–Meier curves start to diverge after 365 days of follow-up. All-cause death within 30 days after intervention in Lombardy occurred in 10 patients (1.3%) after TAVI and in 15 patients (1.9%) after SAVR, [aOR 95%CI 0.66 (0.29–1.50), *p* = 0.3228]. In Puglia, 7 (2.2%) patients in the TAVI cohort and 10 (3.1%) patients in the SAVR cohort died; the trend of risk was similar to that in Lombardy, [aOR 95%CI 0.71 (0.26–1.94), *p* = 0.5087]. 

**Figure 2:**
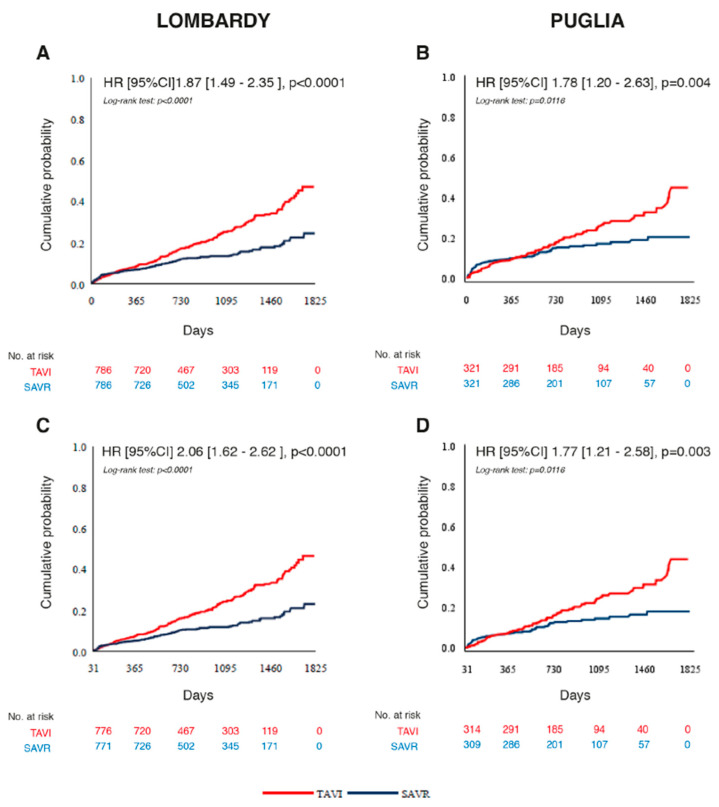
Kaplan–Meier curves of cumulative probability of death by any cause in Lombardy and Puglia. (**A**,**B**) From 0 to 1825 days of follow-up after aortic valve replacement. (**C**,**D**) From 31 to 1825 days follow-up. CI: confidence interval; HR: hazard ratio; SAVR: surgical aortic valve replacement; TAVI: transcatheter aortic valve implantation.

Mortality between 31 and 1825 days after intervention in Lombardy was higher in the TAVI cohort (46.5%) compared to SAVR (23.1%). The adjusted risk for mortality was 2.5-fold higher in the TAVI cohort compared to SAVR (HR 95%CI 2.52 [1.93–3.28], *p* < 0.0001). The cumulative probability of risk is represented in Figure 2. The mortality rates at 2 and 3 years in Lombardy were 17.2% and 25.2%, respectively, in TAVI and 12.0% and 13.6%, respectively, in SAVR. Similar data were found in Puglia: mortality at the end of follow-up was 44.1% in TAVI and 18.1% in SAVR (HR 95%CI 1.74 [1.19–2.55], *p* = 0.0043). The mortality rates at 2 and 3 years were 18.2% and 26.2%, respectively, in TAVI and 15.1% and 17.3%, respectively, in SAVR. The mean DDCI was 5.0 ± 3.0 in TAVI and 5.0 ± 2.9 in SAVR (DDCI values range: 0 to 11), where higher values correspond to higher mortality.

### 3.3. Secondary Outcomes

The sensitivity analysis is reported in the Supplementary Materials as Supplementary Table S1. The secondary outcomes are reported as Supplementary Materials (text, Supplementary Figure S2 and Table S2). These analyses were intended to address the time-related changes in outcomes between TAVI and SAVR, with the intention to stress the procedural vs. long-term differences. At 30 days, patients in the TAVI group required more percutaneous coronary procedures, while patients in the SAVR arm had more surgical coronary revascularization. Patients in the TAVI arm required permanent pace-maker implantation at a higher rate than patients in the SAVR arm.

## 4. Discussion

In our study, after propensity matching, the risk of death from 30 to 1825 post-procedural days was significantly higher in the TAVI group vs. the SAVR group in both Lombardy and Puglia. 

Our results may appear to be conflicting with the existing evidence from randomized control trials showing equivalent long-term outcomes of the two techniques, or even the superiority of TAVI [14,15,16,17,18,19,20]. In high-risk patients, the PARTNER-1 study showed an equivalence of the two techniques, with a 5-year mortality for any cause of 67.8% in TAVI and 62.4% in SAVR [14]. In other high-risk series, Gleason and associates found an all-cause 5-year mortality of 55% in both TAVI and SAVR [15], and Deeb and associates reported a composite outcome of all-cause mortality or stroke that was significantly (*p* = 0.006) lower in TAVI (37.3%) than in SAVR (46.7%) at 3 years of follow-up [16].

In patients at intermediate risk (PARTNER-2), all-cause mortality at 5 years was equivalent in both TAVI and SAVR groups (46.0% and 42.1%, respectively) [17]. Reardon and associates (SURTAVI) [18] reported a composite outcome of all-cause mortality and disabling stroke of 12.6% in TAVI and 14% in SAVR at 2 years of follow-up. Finally, low-risk patients were investigated in the PARTNER-3 trial, finding a 2-year risk of a composite outcome (all-cause death, stroke, cardiovascular re-hospitalization) of 11.5% after TAVI and 17.4% after SAVR (*p* = 0.007) [19]. Another low-risk series found the 5-year risk of a composite outcome not to be statistically different between TAVI and SAVR (38% vs. 36.3%) [20].

We cannot advocate that our patient population was at a low, high, or intermediate risk. It was actually inclusive of non-selected patients, except for in its age range. Based on this factor, our patient population may be considered at an intermediate-for-age risk. In fact, studies on high-risk patients report a mean age around 83 years [14,16], those on intermediate-risk patients a mean age around 80 years [17,18] and those on low-risk patients a mean age around 73 years [19]. Given these figures, our patient population with a mean age of 76 years can be defined as being at intermediate risk for the age criterion, and this is supported by the morbidity-related DDCI, which is about 50% of the range. 

In these “intermediate-for-age-risk patients”, we observed a superiority of SAVR vs. TAVI in terms of 5-year all-cause mortality. The mortality rate in TAVI patients (45%) is in agreement with that reported in trials focused on intermediate-risk patients [17]. Conversely, the 5-year mortality risk for SAVR (20%) is considerably lower than that reported in the same trial [17]. At 2 years of follow-up, mortality in SAVR (11%) is lower than that reported in other trials on intermediate-risk patients [18]. Isolated SAVR is probably the cardiac surgery procedure with a lower mortality risk. A recent report on more than 40,000 low-risk cases showed a 5-year mortality rate of 7.1% [21], which is considerably inferior to what has been reported in RCTs on low-risk patients undergoing SAVR or TAVI. In a low-risk patient population randomized to SAVR or TAVI, the SAVR arm had a 5-year mortality rate of 28% [20], four times higher than what is considered the benchmark [21]. Therefore, in a real-world scenario, the surgical results are better than in randomized controlled trials. It could be that the randomized studies were conducted mainly in institutions with a higher expertise in TAVI than in SAVR; but other factors may be responsible for the better results of the surgery in recent years. Actually, surgery has made great advances, especially with the introduction of self-expandable aortic valves, mini-sternotomy access, minimally invasive cardiopulmonary bypass and other innovations. So, it can be concluded that the comparison between SAVR and TAVI is a fair one if the “best possible TAVI” is compared to the “best possible SAVR”.

There is other evidence that in real-world data the long-term outcomes are better in SAVR than in TAVI. 

A recent meta-analysis [22] that pooled six randomized controlled trials (two for high-risk, two for intermediate-risk and two for low-risk patients) published between 2015 and 2019 [14,16,17,18,19,20]. In the study, the authors found that, at 40 months of follow-up, there was a higher mortality in TAVI vs. SAVR (HR 1.31, 95% CI 1.01–1.68). There are other recent analyses based on long-term mortality. At the Society of Cardiac Surgeons meeting early in 2024, Malas and associates analyzed the administrative data of young patients (≤60 years) in a propensity-matched study [23]. They found a 5-year mortality of 3.3% in SAVR and 11.3% in TAVI (*p* = 0.001). Finally, the Austrian Registry [24] recently published an analysis where the risk of mortality at 5-year follow up was higher in TAVI than in SAVR (hazard ratio 2.4) after correction for potential confounders. This effect was even more pronounced in the segment of population aged 65–75 years old, that is, exactly the area where the US and the European Guidelines diverge. It is reasonable to conclude that, when follow-up is extended up to 5 years, the mortality risk is reduced in SAVR vs. TAVI, whereas at 1 year the outcomes are equivalent [25] or even in favor of TAVI. 

There are limitations in our study. Some pre-procedural variables are missing (risk score stratification according to EuroSCORE II and/or STS-PROM, and others like left ventricular ejection fraction). This may impair the between-group comparability. However, the propensity score matching process did consider as many as 18 pre-procedural factors, the great majority of those composing the EuroSCORE II scale, and the correct matching of the two groups was confirmed by the DDCI. Another possible limitation is that patient selection based on administrative codes (ICD-9) may introduce the (rare) risk of miscoding. However, this may be a concern for secondary outcomes only, whereas mortality is retrieved from the anagraphic registries and therefore is free from any bias. The strength of our study is the inclusion of all cardiac surgery institutions and patients in the two regions considered, thus minimizing the selection bias that is unavoidable in clinical trials.

In conclusion, given the limitations of retrospective analysis, our real-world data suggest that in the age range 65–80 years the outcomes of SAVR may be more favorable than those of TAVI.

## Figures and Tables

**Table 1:** Characteristics at entry into cohort of subjects undergoing TAVI and SAVR in Lombardy and Puglia.

	Lombardy				Puglia			
Variable	TAVI n = 1753	SAVR n = 3537	*p* Value	SMD	TAVI n = 540	SAVR n = 1491	*p* Value	SMD
**Age,** mean ± SD	76.3 ± 3.5	73.4 ± 4.3	<0.0001	0.77	76.5 ± 3.6	73.6 ± 4.2	<0.0001	0.72
Median [Q1–Q3]	77 (74–79)	74.0 (70–77)	<0.0001		78 (74–79)	74 (70–78)	<0.0001	
Min–Max	(65–80)	(65–80)			(65–81)	(65–81)		
**Sex**								
Women. n (%)	815 (46.5)	1.339 (37.9)	<0.0001	0.18	295 (54.6)	591 (39.6)	<0.0001	0.08
Men. n (%)	938 (53.5)	2.198 (62.1)			245 (45.4)	900 (60.4)		
**Two-year period**								
2018–2019. y	783 (44.7)	2.083 (58.9)	<0.0001	−0.29	139 (43.3)	133 (41.4)	<0.0001	0.04
2020–2021. y	970 (55.3)	1.454 (41.1)			182 (56.7)	188 (58.6)		
**Comorbidities of interest (in previous 5 years),** n (%)								
Cerebrovascular disease	179 (10.2)	218 (6.2)	<0.0001	0.15	79 (14.6)	116 (7.8)	<0.0001	0.22
Myocardial infarction	210 (12.0)	195 (5.5)	<0.0001	0.23	67 (12.4)	96 (6.4)	<0.0001	−0.21
Other coronary disease	572 (32.6)	580 (16.4)	<0.0001	0.38	189 (35.0)	257 (17.2)	<0.0001	0.41
Heart failure	570 (32.5)	525 (14.8)	<0.0001	0.43	182 (33.7)	240 (16.1)	<0.0001	0.42
Atrial fibrillation	355 (20.3)	356 (10.1)	<0.0001	0.29	130 (24.1)	195 (13.1)	<0.0001	0.29
Percutaneous transluminal coronary angioplasty	390 (22.2)	270 (7.6)	<0.0001	0.42	102 (18.9)	75 (5.0)	<0.0001	0.44
Coronary artery bypass surgery	29 (1.7)	8 (0.2)	<0.0001	0.15	10 (1.8)	0 (0.0)	<0.0001	0.19
Previous aortic valve replacement	6 (0.3)	9 (0.3)	0.59	0.02	8 (1.5)	6 (0.4)	0.009	0.11
Device therapy	144 (8.2)	124 (3.5)	<0.0001	0.20	36 (6.7)	34 (2.3)	<0.0001	0.21
Peripheral artery disease	113 (6.4)	79 (2.2)	<0.0001	0.21	39 (7.2)	40 (2.7)	<0.0001	0.21
Pulmonary embolia	15 (0.9)	17 (0.5)	0.0977	0.05	3 (0.6)	2 (0.1)	0.009	0.09
Renal disease	210 (12.0)	103 (2.9)	<0.0001	0.35	78 (14.4)	61 (4.1)	<0.0001	0.36
Chronic obstructive pulmonary disease	262 (14.9)	203 (5.7)	<0.0001	0.31	110 (20.4)	175 (11.7)	<0.0001	0.24
Cancer	250 (14.3)	309 (8.7)	<0.0001	0.17	60 (11.1)	120 (8.0)	0.03	0.11
Diabetes	665 (37.9)	841 (23.8)	<0.0001	0.31	219 (40.6)	419 (28.1)	<0.0001	0.27

*p* value: Chi-square test applied for categorical variables and two-sample Wilcoxon test applied for numeric variables. SMD: standardized mean difference.

**Table 2:** Baseline characteristics in post-propensity-matched cohorts of subjects undergoing TAVI and SAVR in Lombardy and Puglia.

	Lombardy				Puglia			
Variable	TAVI n = 786	SAVR n = 786	*p* Value	SMD	TAVI n =321	SAVR n = 321	*p* Value	SMD
**Age, mean** **±** **SD**	75.5 ± 3.9)	75.8 ± 3.5	0.37	0.09	75.9 ± 3.8)	75.8 ± 3.6)	0.88	**0.03**
**Median [Q1–Q3]**	76 (73–79)	77 (74–79)	0.37	0.02	77 (74–79)	76.8 (73–79)	0.88	
**Min–Max**	(65–80)	(65–80)		−0.07	(65–81)	(65–81)		
**Sex**								
**Women. n (%)**	346 (44.0)	368 (46.8)	0.27	−0.06	166 (51.7)	153 (47.7)	0.31	**0.08**
**Men. n (%)**	440 (56.0)	418 (53.2)			155 (48.3)	168 (52.3)		
**Two-year period**								
**2018–2019. y**	411 (52.3)	403 (51.3)	0.69	0.02	139 (43.3)	133 (41.4)	0.63	**0.04**
**2020–2021. y**	375 (47.7)	383 (48.7)			182 (56.7)	188 (58.6)		
**Comorbidities of interest (in previous 5 years), n (%)**								
**Cerebrovascular disease**	87 (11.1)	70 (8.9)	0.15	0.07	41 (12.8)	37 (11.5)	0.63	**0.04**
**Myocardial infarction**	91 (11.6)	74 (9.4)	0.16	0.07	30 (9.3)	34 (10.6)	0.60	**−0.04**
**Other coronary disease**	223 (28.4)	218 (27.7)	0.78	0.01	91 (28.3)	88 (27.4)	0.79	**0.00**
**Heart failure**	219 (27.9)	204 (26.0)	0.39	0.04	86 (26.8)	94 (29.3)	0.48	**−0.06**
**Atrial fibrillation**	135 (17.2)	147 (18.7)	0.43	−0.04	71 (22.1)	69 (21.5)	0.85	**0.01**
**Percutaneous transluminal coronary angioplasty**	141 (17.9)	129 (16.4)	0.42	0.04	40 (12.5)	38 (11.8)	0.80	**0.02**
**Coronary artery bypass surgery**	10 (1.3)	6 (0.8)	0.31	0.05	0 (0.0)	0 (0.0)	-	**-**
**Previous aortic valve replacement**	4 (0.5)	3 (0.4)	1.00	0.02	3 (0.9)	2 (0.6)	0.65	**0.04**
**Device therapy**	49 (6.2)	47 (6.0)	0.83	0.01	15 (4.7)	20 (6.2)	0.38	**−0.07**
**Peripheral artery disease**	44 (5.6)	34 (4.3)	0.25	0.06	17 (5.3)	17 (5.3)	1.00	**0.00**
**Pulmonary embolia**	7 (0.9)	2 (0.3)	0.18	0.08	1 (0.3)	0 (0.0)	0.32	**0.14**
**Renal disease**	65 (8.3)	55 (7.0)	0.34	0.05	24 (7.5)	29 (9.0)	0.47	**−0.05**
**Chronic obstructive pulmonary disease**	100 (12.7)	84 (10.7)	0.21	0.06	56 (17.4)	55 (17.1)	0.92	**0.01**
**Cancer**	103 (13.1)	113 (14.4)	0.47	−0.04	31 (9.7)	22 (6.8)	0.20	**0.11**
**Diabetes**	**269 (34.2)**	**270 (34.4)**	**0.96**	**−0.003**	**112 (34.9)**	**124 (38.6)**	**0.33**	**−0.08**

*p* value: Chi-square test applied for categorical variables and two-sample Wilcoxon test applied for numeric variables. SMD: standardized mean difference.

## Data Availability

Original data are available from the Istituto Mario Negri upon reasonable request.

## Supplementary Materials

The following supporting information can be downloaded at: https://www.mdpi.com/article/10.3390/jcm14103471/s1, Figure S1: Pre and post matching standardized mean difference of demographics and comorbidities; Table S1: Risk of all-cause mortality in TAVI versus SAVR in the original study cohort and in that studied for sensitivity analysis; Figure S2: Kaplan-Meier curves of cumulative probability of events 31 to 1825 days follow-up in Lombardy and Puglia region related to: (A) and (B) events related to valve replacement procedures; (C) and (D) cardiovascular events; (E) and (F) non-cardiovascular events. CI: confidence interval; HR: hazard ratio; SAVR: surgical aortic valve replacement TAVI: transcatheter aortic valve implantation. Table S2: Secondary outcomes in the propensity score matched population.

## Appendix A

Comorbidities of interest (in the previous 5 years).

**#**	**Comorbidities**	**Patients’ Record From**	**Classification**	**ICD-9CM Code**
1	**Cerebrovascular disease**	Admission	ICD9	from 430 * to 437 *
		Admission	Procedures	3811, 3812
		ER	ICD9	from 430 * to 437 *
2	**Myocardial infarction**	Admission	ICD9	410 *, 412 *
		ER	ICD9	410 *, 412 *
3	**Other coronary disease**	Admission	ICD9	413 *, 414 *
		ER	ICD9	413 *, 414 *
4	**Heart failure**	Admission	ICD9	428 *, 39891, 40201, 40211, 40291, 40401, 40403, 40411, 40413, 40491, 40493
		ER Admission	ICD9	428 *, 39891, 40201, 40211, 40291, 40401, 40403, 40411, 40413, 40491, 40493
5	**Atrial fibrillation**	ER Admission	ICD9	42731, 42732
			ICD9	42731, 42732
6	**Percutaneous transluminal coronary angioplasty**	Admission	Procedures	from 0040 to 0048, 0066, 3606, 3607, 3609
7	**Coronary artery by-pass surgery**	Admission	Procedures	361, 362, 363, da 3610 a 3619, da 3631 a 3634, 3639
8	**Previous aortic valve replacement**	Admission	Procedures	3521, 3522
9	**Device therapy**	Admission	Procedures	0050, 0051, 0053, 0054, 3762, 3765, 3766, 3767, 3768, 3771, 3774, 3782, 3783, 3785, 3787, 3789, 3794, 3796, 3797, 3798
10	**Peripheral artery disease**	Admission	ICD9	2507 *, 4402 *, 4403, 44030, 44032, 44381
10 11	**Peripheral artery disease** **Pulmonary embolia**	Admission	Procedures ICD9	3808, 3818, 3925, 3926, 3929, 3950, 39902507 *, 4402 *, 4403, 44030, 44032, 44381
10 11 11 12	**Peripheral artery disease** **Pulmonary embolia** **Renal disease** **Pulmonary embolia**	ER Admission	ICD9 Procedures	2507 *, 4402 *, 4403, 44030, 44032, 44381 3808, 3818, 3925, 3926, 3929, 3950, 3990
		ER Admission	ICD9	4151, 41511, 415192507 *, 4402 *, 4403, 44030, 44032, 44381
		Admission	ICD9	585, V451, V561, V562, V563 *, 581814151, 41511, 41519
12 13	**Renal disease** **Chronic obstructive pulmonary disease** **Respiratory insufficiency**	Admission	Procedures ICD9	3895, 3927, 3942, 3943, 3995, 5498585, V451, V561, V562, V563 *, 58181
12 13 13 14	**Renal disease** **Chronic obstructive pulmonary disease** **Respiratory insufficiency** **Chronic obstructive pulmonary disease** **Cancer**	ER Admission	ICD9 Procedures	585, V451, V561, V562, V563 *, 581813895, 3927, 3942, 3943, 3995, 5498
		ER Admission	ICD9	491 *, 492 *, 493 *, 494 *, 496 *, 51881, 51883, 51884585, V451, V561, V562, V563 *, 58181
		ER Admission	ICD9	491 *, 492 *, 493 *, 494 *, 496 *, 51881, 51883, 51884491 *, 492 *, 493 *, 494 *, 496 *, 51881, 51883, 51884
13 14 14 15	**Chronic obstructive pulmonary disease/Respiratory insufficiency** **Cancer** **Cancer** **Diabetes**	Admission ER	ICD9	from 140 * to 165 *, from 170 * to 208 *, from 210 * to 239 *, 2592491 *, 492 *, 493 *, 494 *, 496 *, 51881, 51883, 51884
		ER Admission	ICD9	from 140 * to 165 *, from 170 * to 208 *, from 210 * to 239 *, 2592, from 140 * to 165 *, from 170 * to 208 *, from 210 * to 239 *, 2592
14 15 15	**Cancer** **Diabetes** **Diabetes**	Drugs ER	ATC ICD9	At least 2 A10 * prescriptions on two different dates in the 365 days preceding the index intervention from 140 * to 165 *, from 170 * to 208 *, from 210 * to 239 *, 2592
		ExemptionsDrugs	ATC	At least one record with exemption code 013, exemption start date before the index event and exemption end date missing or after the observation startAt least 2 A10 * prescriptions on twodifferent dates in the 365 days preceding the index intervention
15	**Diabetes**	Exemptions		At least one record with exemption code 013, exemption start date before the index event and exemption end date missing or after the observation start
* Only the main category codes are shown in the table, but all subcategories are included in the selection.				

## Appendix B

Secondary endpoints.

**Diagnosis**	**ICD-9CM Code**		
**Heart failure**	428,x; 398,91; 402,01; 402,11; 402,91; 404,01; 404,03; 404,11; 404,13; 404,91; 404,93		
**Atrial fibrillation**	427,31; 427,32		
**Unstable angina**	411; 411,0; 411,1; 411,8; 411,81; 411,89		
**Myocardial infarction**	410,x; 412		
**Percutaneous transluminal coronary angioplasty** **Coronary artery by-pass surgery**	36,1,x; 36,15; 36,2; 36,3 36,0,x 00,40 al 00,48; 00,66		
**Stroke [ischemic, haemorragic]**	433,01; 433,11; 433,21; 433,31; 433,81; 433,91; 434,01; 434,91; 430; 431; 432; 432,0; 432,1; 432,9		
**Definitive pacemaker implantation**	00,50; 37,71; 37,72; 37,82; 37,83		
**Renal insufficiency**	585,x; V451; 39,95; 54,98; V561; V562; V563; V563,1; V563,2; 38,95; 39,27; 39,42; 39,43; 581,81		
**Bacterial endocarditis**	421,x		
**Aortic dissection**	4410 *		
**Senile dementia**	290,2; 290,3		
**Vascular dementia**	290,4; 290,41; 290, 42; 290,43		
**Mediastinitis**	519,2		
**Respiratory failure**	518,81; 518,83; 518,84		
**Gram- endotoxic septic shock**	785,52		
**New valve replacement**	35,21; 35,22		
**Events related to the aortic prosthetic valve**	996,02	Mechanical complications from cardiac valve prosthesis	
	996,61		Infection and inflammatory reaction from cardiac prostheses, implants and grafts
	996,71		Other complications from aortic prosthetic valve
* Only the main category codes are shown in the table, but all subcategories are included in the selection.			
