# Supplementary material for: When Real-World Outcomes Do Not Meet the Results of Clinical Trials: Transfemoral Transcatheter vs. Surgical Aortic Valve Replacement in an Intermediate-Age Population (The Outstanding Italy Study)
Source: J Clin Med. 2025 May 15;14(10):3471. doi: 10.3390/jcm14103471 (PMC12539603; doi:10.3390/jcm14103471)
Supplement: Supplementary file 1 [file jcm-14-03471-s001.zip › jcm-3548915-supplementary.pdf]

Supplementary figure 1

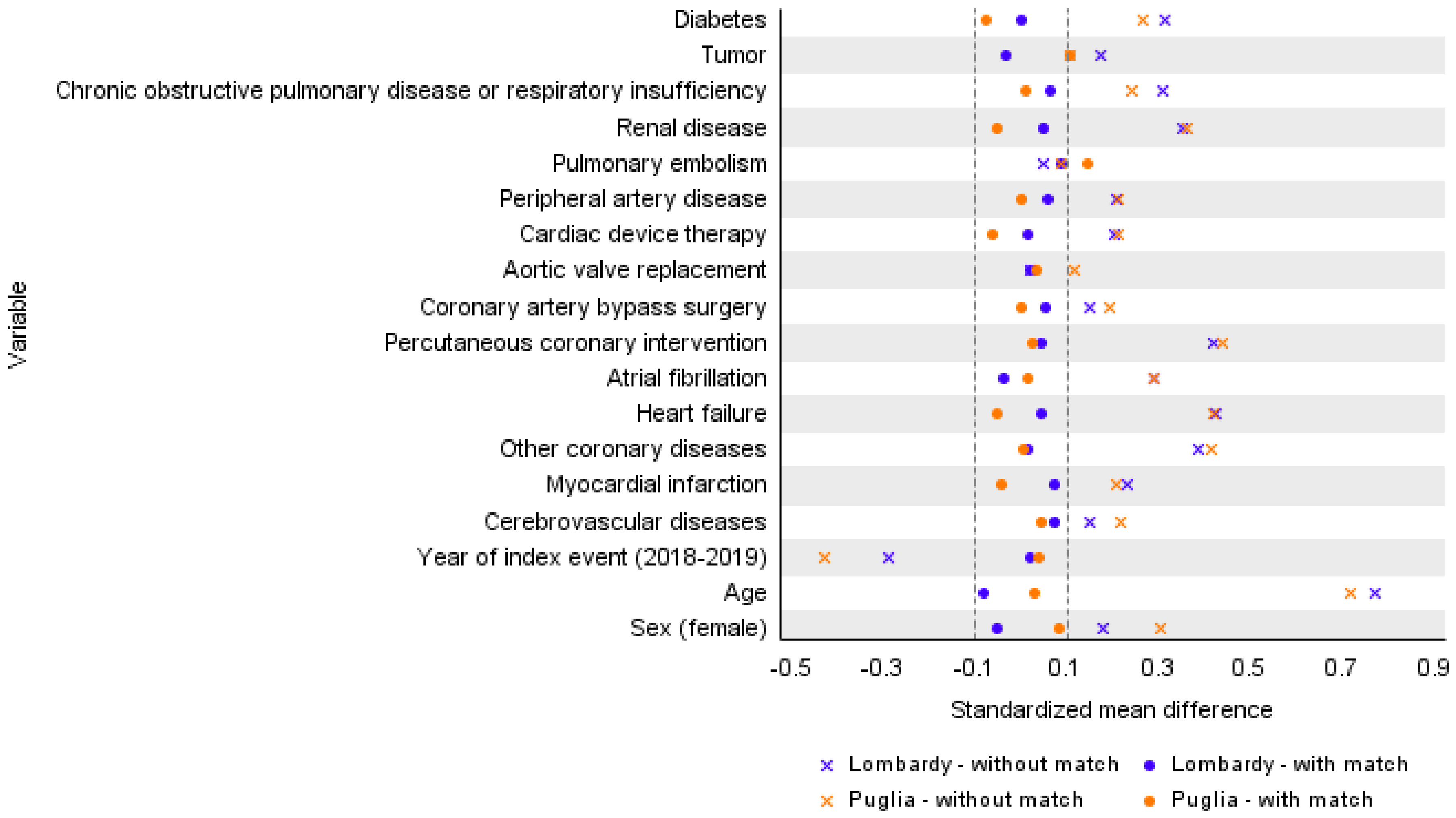

Supplementary Figure 1. Pre and post matching standardized mean difference of demographics and comorbidities.

Supplementary material 1

Sensitivity analysis

The results from the sensitivity analysis showed that the mean DDCI index was 5.0±3.0 in TAVI 5.0±2.9 in SAVR. The rate of hospitalization or visit in the emergency department for COVID-19 during follow-up was 8.4% in TAVI and 8.3% in SAVR.

Total mortality rate at different time points was similar to that described in the original cohorts: mortality at the end of follow-up was, 41.7% in TAVI, 28.1% in SAVR and at 2 and 3 years was 15.3% and 22.8%, respectively in TAVI and 13.5% and 15.9% in SAVR. The adjusted risk for mortality in TAVI compared with SAVR in the overall cohort and in subjects with and without COVID-19 are reported in **Supplementary Table 1.**

Supplementary Table 1. Risk of all-cause mortality in TAVI versus SAVR in the original study cohort and in that studied for sensitivity analysis

	Lombardy cohort n=1572		Puglia cohort n=642		Sensitivity analysis n=1866*	
All-cause mortality	aHR or aOR (95% CI)	P value	aHR or aOR (95% CI)	P value	aHR or aOR (95% CI)	P value
0-30 days	0.66 (0.29-1.50)	0.33	0.71 (0.26-1.94),	0.51	0.52 (0.26-1.07)	0.078
> 30 days	2.52 (1.93-3.28)	<0.0001	1.74 (1.19-2.55)	0.0043	1.74 (1.39-2.18)	<0.0001
COVID-19 yes (n=155)	-	-	-	-	2.04 (1.16-3.60)	0.014
COVID-19 no (n=1711)	-	-	-	-	1.53 (1.21-1.93)	0.0003

aHR, adjusted hazard ratio; aOR, adjusted odds ratio; CI, confidence interval. aOR was calculated for 0-30 days mortality
* After the inclusion of DDCI and hospital admission for COVID-19, the number of patients increased.

Supplementary material 2

Secondary outcomes in propensity-score matched cohorts

The incidence of events related to the prosthetic valve was the same in the TAVI and SAVR cohorts in Lombardy (0.1%) and in Puglia (0.6%) (**Supplementary table 2**) in the first 30 post-procedural days. From 31 to 1,825 days after the procedure, the incidence of events related to the prosthetic valve in Lombardy was 3.1% in TAVI, 3.6 % in SAVR, (cHR [95%CI] of 0.91 [0.48 -1.75], p=0.78) and in Puglia 3.6% and 4.4%, (cHR [95%CI] of 0.55 [0.16 -1.84], p=0.33) (**Supplementary Table 2, Supplementary Figure 2**).

Early (within 30 days) cardiovascular events occurred less frequently in the TAVI cohort compared to SAVR both in Lombardy (27.2 % vs 34.1%, aOR [95% CI]: 0.56 [0.47-0.66], p<0.0001) and Puglia (24.0 % in TAVI vs 32.7% in SAVR, aOR [95% CI]: 0.65 [0.46-0.92], p<0.0145).

A pacemaker implantation was performed in Lombardy in 12.0% and 3.4% patients in TAVI and SAVR cohort respectively (p <0.0001); 13.4% and 4.7% in Puglia (p=0.0001).

Between 31 to 1,825 days the incidence of hospitalizations for cardiovascular reasons was similar in TAVI and SAVR cohorts in Lombardy and Puglia.

The most frequent causes for cardiovascular hospitalizations were heart failure and stroke both in Lombardy and Puglia (**Supplementary Table 2**).

Non -cardiovascular events

Early (within 30 days) non-cardiovascular events after AVR were 1.9% in TAVI cohort and 1.3% in SAVR in Lombardy while in Puglia was 3.7% in TAVI and 3.4% in SAVR (**Supplementary Table 2**). From 31 to 1,825 days the incidence of a first hospitalization for non-cardiovascular reasons was 18.3% in the TAVI cohort and 10.8% in SAVR (cHR [95% CI], 1.95 [1.31-2.91], p<0.001) in Lombardy and 9.7% in TAVI and 7.6% in SAVR in Puglia (**Supplementary Table 2**, **Supplementary Figure 2**).

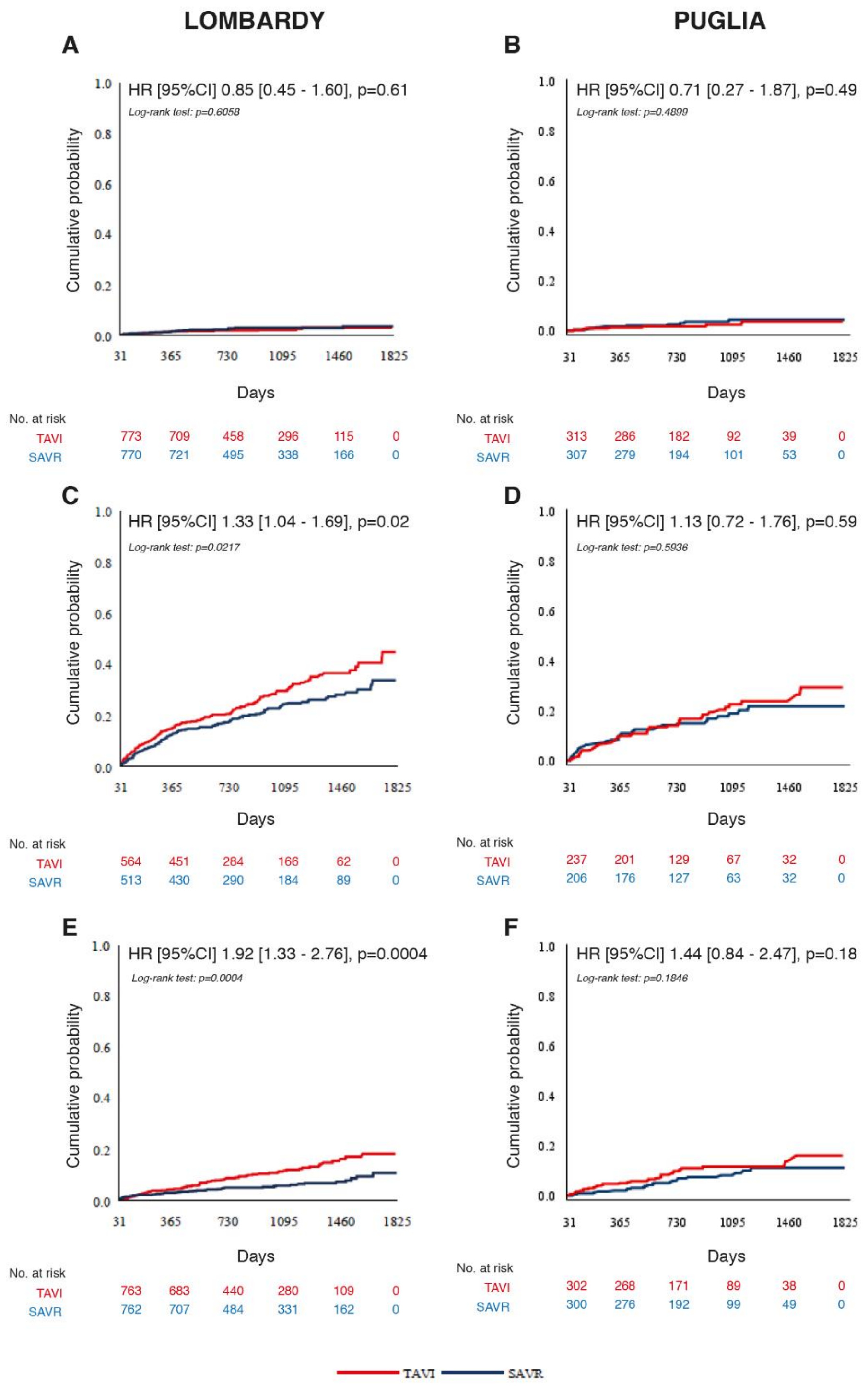

Supplementary Figure 2. Kaplan-Meier curves of cumulative probability of events 31 to 1,825 days follow-up in Lombardy and Puglia region related to: A) and B) events related to valve replacement procedures; C) and D) cardiovascular events; E) and F) non-cardiovascular events. CI: confidence interval; HR: hazard ratio; SAVR: surgical aortic valve replacement TAVI: transcatheter aortic valve implantation.

Supplementary Table 2. Secondary outcomes in the propensity score matched population.

Type of Event	Lombardy				Puglia			
	Thirty days follow-up		Thirty-one to 1825 days follow-up		Thirty days follow-up		Thirty-one to 1825 days follow-up	
	TAVI n=786	SAVR n=786n	TAVI n=786	SAVR n=786	TAVI n=321	SAVR n=321n	TAVI n=321	SAVR n=321n
**Related to AVR**	n (%)	n (%)	n (%)	n (%)	n (%)	n (%)	n (%)	n (%)
Bacterial endocarditis	1 (0.1)	-	14 (1.8)	17 (2.2)	-	-	4 (1.2)	4 (1.2
Aortic dissection	-	-	1 (0.1)	-	-	-	-	2 (0.6)
Mediastinitis	-	-	-	-	-	-	-	
New valve replacement	1 (0.1)	-	4 (0.5)	7 (0.9)	1 (0.3)	-	3 (0.9)	5 (1.6)
Prosthetic valve related event	1(0.1)	1 (0.1)	4 (0.5)	5 (0.6)	-	2 (0.6)	2 (0.6)	2 (0.6)
**Cardiovascular**								
Heart failure	25 (3.2)	9 (1.1)	104 (13.2)	83 (10.6)	4 (1.2)	6 (1.9)	29 (9.0)	25 (7.8)
Atrial fibrillation	6 (0.8)	1 (0.1)	37 (4.7)	49 (6.2)	1(0.3)	6 (01.9)	3 (0.9)	5 (1.6)
Unstable angina	3 (0.4)	3 (0.4)	15 (1.9)	3 (0.4)	-	1 (0.3)	3 (0.9)	6 (1.9)
Myocardial infarction	2 (0.3)	4 (0.5)	28 (3.6)	10 (1.3)	2 (0.6)	1 (0.3)	8 (2.5)	7 (2.2)
Percutaneous transluminal coronary angioplasty	108 (13.7)	17 (2.2)	35 (4.5)	28 (3.6)	27 (8.4)	6 (1.9)	7 (2.2)	7 (2.2)
Coronary artery by-pass surgery	1(0.1)	223 (28.4)	1 (0.1)	2 (0.3)	1(0.3)	76 (23.7)	-	1 (0.3)
Stroke	4 (0.5)	3 (0.4)	26 (3.3)	23 (2.9)	-	-	15 (4.7)	8 (2.5)
Definitive pacemaker implantation	94 (12.0)	27 (3.4)	32 (4.1)	22 (2.8)	43 (13.4)	15 (4.7)	12 (3.7)	13 (4.0)
**Non-cardiovascular**								
Gram- endotoxic septic shock	-	-	1 (0.1) -	3 (0.4)	1 (0.3)	2 (0.6)	4 (1.2)	2 (0.6)
Renal failure	11 (1.4)	10 (1.3)	26 (3.3)	15 (1.9)	9 (2.8)	10 (3.1)	12 (3.7)	4 (1.2)
COPD/Respiratory insufficiency	4 (0.5)	-	55 (7.0)	34 (4.3)	2 (0.6)	1 (0.3)	21 (6.5)	17 (5.3)

AVR, aortic replacement, COPD, chronic obstructive pulmonary disease
